# Detection of Chronic Musculoskeletal Pain Using Voice Characteristics

**DOI:** 10.1109/JTEHM.2025.3553892

**Published:** 2025-03-24

**Authors:** Masakazu Higuchi, Toshiko Iidaka, Chiaki Horii, Gaku Tanegashima, Hiroyuki Oka, Hiroshi Hashizume, Hiroshi Yamada, Munehito Yoshida, Sakae Tanaka, Noriko Yoshimura, Mitsuteru Nakamura, Shinichi Tokuno

**Affiliations:** Department of BioengineeringGraduate School of EngineeringThe University of Tokyo 13143 Tokyo 113-8656 Japan; Department of Preventive Medicine for Locomotive Organ Disorders22nd Century Medical and Research CenterThe University of Tokyo 13143 Tokyo 113-8655 Japan; Department of Orthopaedic Surgery, Sensory and Motor System MedicineGraduate School of MedicineThe University of Tokyo 13143 Tokyo 113-8655 Japan; Department of Medical Research and Management for Musculoskeletal Pain22nd Century Medical and Research CenterThe University of Tokyo 13143 Tokyo 113-8655 Japan; Department of Orthopedic SurgeryWakayama Medical University School of Medicine Wakayama 641-8509 Japan; Graduate School of Health InnovationKanagawa University of Human Services 38284 Kawasaki 210-0821 Japan

**Keywords:** Chronic musculoskeletal pain, logistic regression analysis, voice characteristics

## Abstract

Physical pain, particularly musculoskeletal pain, negatively impacts the activities of daily life and quality of life of elderly people. Because pain is a subjective sensation and there are no standard assessment procedures to detect pain, we attempted to quantitatively determine the actual state of chronic pain caused by musculoskeletal organs and related factors based on questionnaires. First, we studied techniques for diagnosing diseases by monitoring the involuntary characteristics of the voice. Then, we applied the technique based on voice characteristics and proposed a voice index to detect chronic musculoskeletal pain. The voice index was derived based on the assumption that physiological changes due to chronic musculoskeletal pain also affect the vocal cords. Subjects in this study were adults, 65 years of age or older, with chronic pain in the musculoskeletal system (lumbar and/or knees). A large-scale population-based cohort study was conducted in 2019. Voice characteristics were extracted from the recorded voices of the subjects, and the characteristics with similar properties were organized into several principal components using principal component analysis. The principal components were further combined using logistic regression analysis to propose a voice index that discriminates between normal subjects and subjects suffering from chronic musculoskeletal pain. A discrimination accuracy of approximately 80% was obtained using the dataset corresponding to the participants with knee pain only, and a discrimination accuracy of approximately 70% was obtained during cross-validation of the same dataset. The proposed voice index may serve as a novel tool for detecting chronic musculoskeletal pain. Clinical impact: The voice-based pain detection holds clinical significance owing to its noninvasive nature, ease of administration, and potential to efficiently assess large populations within a short time frame.

## I. Introduction

Chronic pain, particularly musculoskeletal pain, is an important symptom of musculoskeletal disease. According to the 2019 National Livelihood Survey results, lumbar pain was recorded the most among males and the second highest among females, while pain in the limbs and joints was recorded as the fifth highest complaint among males and the third highest among females [1]. Therefore, in the third term of *Healthy Japan 21* [2], a target value was set to reduce the proportion of elderly people with leg and lumbar pain (per 1,000 people), and national prevention activities were promoted. However, pain is a subjective sensation, and there is no established standard assessment method. Therefore, determining the actual state of chronic pain caused by musculoskeletal organs and related factors is dependent on questionnaires and responses from subjects during examinations by physicians.

The visual analog scale (VAS) and numerical rating scale (NRS) are widely used in clinical practice to evaluate pain. These self-administered questionnaires are the de facto standards for subjective pain assessment [3]. Pain evaluation scales, based on third-party observations that focus on facial expressions and behaviors, have also been proposed [4]. Although self-administered psychological tests are relatively simple, they cannot eliminate biased reporting [5], in which certain information is selectively under- or over-estimated by the conscious or unconscious minds of the subjects. The problem of biased reporting is particularly serious among elderly people who often do not actively report pain because of pain-related prejudices and their own endurance and stoicism [6]. Physiological markers, such as blood pressure, heart rate, and pupil diameter, have been used to objectively assess pain; however, their accuracy is questionable [7]. By contrast, functional nuclear magnetic resonance imaging and electroencephalography have been used to provide more focused and accurate pain measurements; however, existing pain detection methods are expensive and highly invasive [8], [9].

Based on the assumption that involuntary elements of voice vary with diseases, we studied techniques to objectively measure these elements and detect diseases, such as depression and dementia [10], [11], [12]. Voice analysis is non-invasive, does not require special equipment, and can be easily performed remotely. Furthermore, voice analysis may solve the problem of biased reporting in self-administered psychological tests. In the post COVID-19 era, remote voice analysis has gained significant attention as a screening tool. For example, when stress is applied to the brain, the vocal cords and heart experience its effects through nerves, causing involuntary reactions, such as a strained voice and changes in heart rate. Physical pain is also known to cause physiological changes, such as increased heart rate, disrupted breathing, and sweating [3], all of which may also affect the vocal cords.

As a part of the research on osteoarthritis/osteoporosis against disability (ROAD) study, we manage and track a large population-based cohort study [13]. The ROAD study was established in 2005 as a nationwide prospective study that aimed to elucidate the environmental and genetic backgrounds of bone and joint diseases, especially osteoporosis and osteoarthritis. The ROAD study includes population-based cohort studies from three communities in Japan: (i) an urban region in Itabashi, Tokyo; (ii) a mountainous region in Hidakagawa, Wakayama; and (iii) a coastal region in Taiji, Wakayama. All participants are recruited from the resident registration lists in their communities. Details of the ROAD study are described elsewhere [13]. The fifth survey of the ROAD study was conducted during 2018–2019 in the mountainous and coastal regions. During the fifth survey of the ROAD study in 2019, we conducted a voice survey of participants. The purpose of this study was to identify subjects with chronic musculoskeletal pain by analyzing various characteristics of their voices. A voice index was proposed to selectively identify subjects with chronic musculoskeletal pain.

## II. Methodology

### A. Ethical Considerations

Informed consent was obtained from all participants, and the study was approved by the ethics committees of the University of Tokyo (Nos. 1264 and 1326) and Wakayama Medical University (No. 373).

### B. Large Population-Based Cohort Study

This study utilized data from the fifth survey of the ROAD study. Among the participants, 827 and 1175 subjects were from the mountainous and coastal regions, respectively. For all the subjects, radiographs of both feet were recorded. The subjects were then made to answer an interviewer-administered questionnaire, which included questions on medical history, family history, physical activity, and joint pain.

For each cohort, medical information regarding pain was obtained from experienced physicians. The subjects were questioned about pain in both knees. The following questions were asked: (i) “Did you experience right knee pain on most days (and continuously for at least one day) in the past month, in addition to the current pain?” and (ii) “Did you experience left knee pain on most days (and continuously for at least one day) in the past month, in addition to the current pain?” The subjects who answered “yes” were considered to have knee pain. For identifying subjects with lumbar pain, the following question was asked: “Did you experience lumbar pain on most days (and continuously for at least one day) in the past month, in addition to the current pain?” The subjects who answered “yes” were considered to have lumbar pain.

### C. Subjects

In this study, we targeted older adults, 65 years of age or older, from the coastal region. Subjects were selected from participants of the fifth ROAD study conducted in the coastal area. Only the coastal group was approached for this study because the survey in the mountainous areas had already been completed by the time the voice characteristics-based analysis was designed and implemented. Since aging societies are evolving worldwide [14], the most urgent issue is the evaluation of physical pain to prevent deterioration of their overall health. Physical pain negatively impacts the activities of daily life (ADL) and quality of life (QoL) of elderly people [15], [16]. The definition of the elderly as 65 years of age or older is based on the definition by the World Health Organization of the United Nations.

**FIGURE 1.**
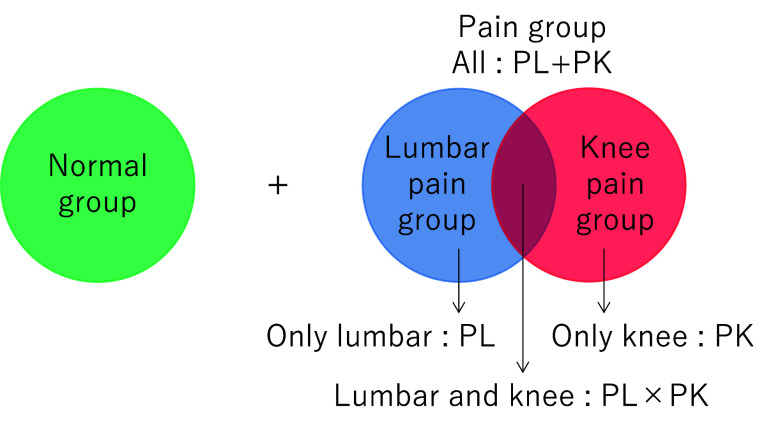
Representation of various pain sub-groups.

Pain examinations were performed on each part of the body of the subjects by an orthopedic physician, as described in Section II-B. Based on the results of the pain examinations, the subjects were divided into two categories: (i) those who did not feel pain in any part of the body in the past month or at the time of the examination (normal group) and (ii) those who felt pain in their lumbar spine, knees, or both in the past month and at the time of the examination (pain group). The pain group was further subdivided into three groups: (i) those who felt pain only in the lumbar spine, (ii) those who felt pain only in knees, and (iii) those who felt pain in both lumbar spine and knees. The pain group and its subgroups (i), (ii), and (iii) are denoted as PL+PK (PL or PK), PL, PK, and PL $\times $PK (PL and PK), respectively, as shown in Fig. 1.

### D. Voice Recording

Voice recordings were performed in a room at the multipurpose center in Taiji, Wakayama. The subjects were asked to read six fixed phrases in Japanese and utter one long vowel sound. Table 1 lists the fixed phrases and the long vowel. Voices were recorded using a portable recorder R-26 (Roland, Japan) and an ME52W pin microphone (OLYMPUS, Japan) at 24-bit / 96 kHz.

**TABLE 1:** The List of the Fixed Phrases and Long Vowel

Type	Japanese Phrase	English Translation
Phrase 1	I-ro-ha-ni-ho-he-to	No meaning; like “a-b-c”
Phrase 2	Honjitsu wa seiten nari	“It is fine today” (standard radio test)
Phrase 3	Garapagosu shotō	Galapagos Islands
Phrase 4	Totemo genki desu	I am very cheerful
Phrase 5	Kinō wa yoku nemuremashita	I slept well yesterday
Phrase 6	Shokuyoku ga arimasu	I have an appetite
Long vowel	A – – –	AH – – –

### E. Voice Analysis

The voices were normalized in volume to compensate for the differences in volume among the voices of the subjects. Only the long vowel sound was used in this analysis. The voice characteristics were extracted and evaluated using the openSMILE software [17], which offers various scripts for automatically calculating different sets of characteristics from voice. The large openSMILE emotion feature set, developed for emotion recognition, was used in this study. Using the voice of a single subject, the computed voice characteristics are as follows:

A) As low-level descriptors, 56 types of acoustic and physical quantities were calculated at the frame level; the quantities include fast Fourier transform (FFT), mel-frequency cepstral coefficients (MFCCs), voiced speech probability, zero-crossing rate, signal energy, and fundamental frequency (F_0_).
B) Using the descriptors from part A), three types of temporal statistics, including moving average, first-order change over time (“delta”), and second-order change over time (“delta-delta”), were calculated.
C) As high-level descriptors, 39 types of statistical functionals, including the mean, maximum, minimum, centroid, quartiles, variance, kurtosis, and skewness, were calculated from the time series in part B).

Therefore, a total of $56\times 3\times 39=6552$ voice characteristics were calculated. Subsequently, a learning algorithm based on statistical methods was applied to the voice characteristics as the training data to construct an index that discriminates between the normal and pain groups.

Our previous studies showed that depression and dementia affect certain voice characteristics of a person [11], [12]. Chronic pain may possibly cause diseases, such as depression and dementia [18], [19]; therefore, to prevent misclassifying pain as a disease, we first excluded voice characteristics that correlate with depression evaluation index values and mini-mental state examination (MMSE) [20] scores. We used our previously proposed vitality index as the depression evaluation index [10]. Voice characteristics that significantly differ between men and women were excluded. The voice characteristics that were effective for discrimination were selected. The discriminative performances of the normal and pain groups for each of the characteristics were evaluated using the area under the curve (AUC) of the receiver operating characteristic (ROC) curve, and the characteristics for the number of subjects were selected in the order of increasing values of AUC. Since some of the selected characteristics were essentially similar to each other, principal component analysis was applied to organize the similar characteristics into several principal components. For the selection of characteristics, matching the number of characteristics to the number of subjects is necessary because if the number of characteristics exceeds the number of subjects, the covariance matrix of the characteristics fall in rank, and principal component analysis cannot be applied.

**FIGURE 2.**
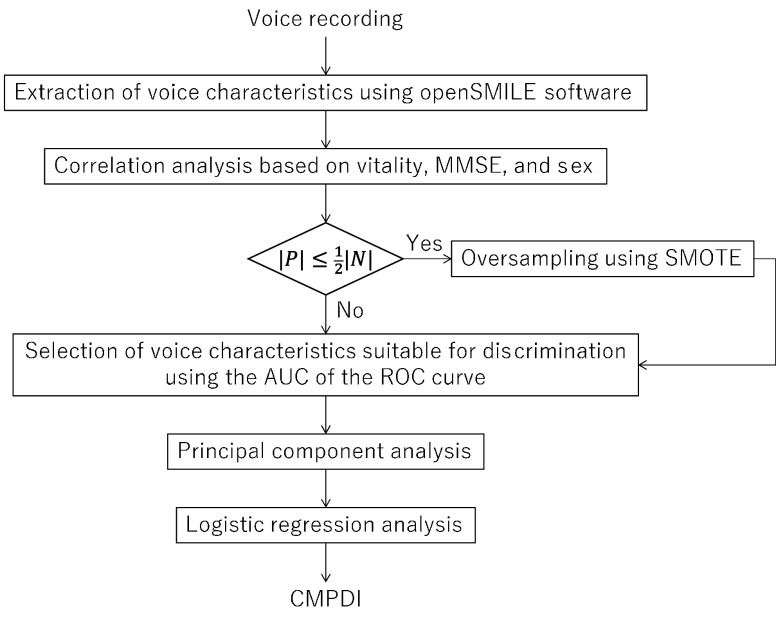
Flowchart of voice analysis ( $|N|$ is the number of samples in the normal group, and $|P|$ is the number of samples in the pain group).

Next, the regularized logistic regression analysis [21] as the learning algorithm was performed, with the obtained principal components as explanatory variables and the pain group as the positive group. In other words, when performing the logistic regression analysis, subjects in the pain and normal groups were assigned positive and negative labels, respectively. Regularization was performed using the L2 norm (ridge regression). The optimal value of the regularization parameter was determined by a grid-search combined with the cross-validation test. The cross-validation test was performed by randomly dividing the data to be trained. Since data division was randomly performed during the cross-validation tests on the training data, the regularization parameter had a different value each time the regression algorithm was executed, and consequently, the regression results were somewhat different each time the algorithm was executed. Therefore, to stabilize the regression results, the regression algorithm was run multiple times (~100 times). The regularization parameters obtained in each run were averaged, and the regression result obtained using the averaged regularization parameter was used as the final regression result. The final regression result was used as the regression coefficient for the principal components. The value of the logistic function, determined using the linear sum of the principal components and regression coefficient as a variable, was used as the chronic musculoskeletal pain detection index (CMPDI). The discrimination threshold for separating the normal and pain groups was defined as the value that minimized the balanced error rate (BER), which is the harmonic mean of the false positive and false negative rates [22]. The flow chart in Fig. 2 shows an outline of the voice analysis experiment. The CMPDI values were obtained to perform the above process for four pairs of groups: (a) normal and PL+PK, (b) normal and PL, (c) normal and PK, and (d) normal and PL $\times $PK groups.

If sampling bias occurs in one of the groups during the regression analysis, the discrimination results may also be biased. In such a case, the obtained discriminant index may have a good accuracy rate (correct discrimination rate); however, the sensitivity (positive detection rate) and specificity (negative detection rate) may be good in one of the cases and poor in the other. In such cases, the synthetic minority oversampling technique (SMOTE) [23] was used to resolve the imbalance. The SMOTE connects a sample point from a small sample group and its neighboring point with a line and randomly generates a data point on that line as an artificial data point. The SMOTE was applied to resolve the data imbalance when the number of subjects in a pain group was less than half of that in the normal group, and vice versa; the groups that did not meet the criterion were considered unbiased. The criterion for applying the SMOTE was met for two pairs of groups: (i) the normal and PK and (ii) normal and PL $\times $PK groups; SMOTE was applied to the data for these groups to eliminate sample imbalance. The performance of each CMPDI was evaluated based on the sensitivity, specificity, accuracy, Welch’s t-test, and *K*-fold cross-validation test. Sensitivity represents the positive detection rate (true positive rate) from the positive group and specificity represents the negative detection rate (true negative rate) from the negative group. Accuracy represents the correct rate corresponding to both positive and negative groups. In Welch’s t-test, the difference of distributions in the CMPDI values between the normal and pain groups was evaluated. In *K*-fold cross-validation test, the training dataset was randomly partitioned into *K* equal sized sub-datasets, referred to as “folds”. Of the *K* subsamples, a single subsample was retained as the validation data for testing the index, and the remaining $K-1$ subsamples were used as training data. The cross-validation process was then repeated *K* times, with each of the *K* subsamples used exactly once as the validation data, and the obtained results were accumulated to create a final result for the training dataset.

Statistical analysis was conducted using free source software R (version 4.0.2, an official part of the Free Software Foundation’s GNU project) [24].

## III. Results

### A. Subjects

Among 1175 subjects, 1167 completed the pain-related interview with an orthopedic physician; voice recordings were collected for 1152 subjects. Among 1152 subjects, 580 were 65 years of age or older, and 512 reported chronic pain. Among 512 subjects, 244 were in the normal group, and 268 were in the pain group (PL+PK group; Fig. 1). Among 268 subjects in the pain group, 143, 54, and 71 subjects were in the PL, PK, and PL $\times $PK groups, respectively. Other details of the subjects are presented in Table 2. (For reference, the attributes of the normal and pain groups for participants under 65 years of age are presented in Table 9 in the Appendix.)

**TABLE 2:** Sex Ratios and Mean Values (Standard Deviations) of the Attributes for Each Group

	Normal Group	PL or PK	PL only	PK only	PL and PK	Difference between groups
	Total number, 244	Total number, 268	Total number, 143	Total number, 54	Total number, 71	(p value)
Men/Women	84/160	72/196	54/89	10/44	8/63	***
Age (years)	73.5 (6.4)	73.2 (5.7)	72.4 (5.6)	73.8 (5.2)	74.5 (5.9)	0.12
Weight (kg)	54.7 (11.5)	55.5 (10.7)	56.3 (11.2)	53.8 (9.2)	55.3 (10.8)	0.58
Height (cm)	156.7 (8.9)	155.1 (8.6)	157.3 (9.0)	153.5 (7.7)	151.8 (7.1)	***
BMI (kg/m^2^)	22.2 (3.5)	23.0 (3.4)	22.6 (3.5)	22.8 (2.9)	23.9 (3.6)	**

BMI = body mass index, ** Significantly different (p < 0.01), *** Significantly different (p < 0.001)

**TABLE 3:** Types and Numbers of Selected Characteristics From Each Dataset

	PL or PK vs Normal	PL vs Normal	PK vs Normal	PL and PK vs Normal
Amplitude energy related	14 (2.7%)	12 (3.1%)	3 (0.7%)	2 (0.4%)
F_0_-related	9 (1.8%)	9 (2.3%)	8 (1.7%)	7 (1.5%)
Spectral power related	220 (43.0%)	166 (42.9%)	95 (20.7%)	223 (48.8%)
Mel-frequency spectrum power related	155 (30.3%)	116 (30.0%)	295 (64.1%)	146 (31.9%)
MFCC-related	92 (18.0%)	68 (17.6%)	49 (10.7%)	63 (13.8%)
Zero crossing rate related	16 (3.1%)	12 (3.1%)	7 (1.5%)	15 (3.3%)
Voiced sound probability related	6 (1.2%)	4 (1.0%)	3 (0.7%)	1 (0.2%)

The percentages (in parentheses) indicate the ratio of the listed characteristics to the total number of selected characteristics from each dataset.

**TABLE 4:** Confusion Matrix When Discriminating Between PL+PK and Normal Groups Using the Discrimination Threshold of ${\text {CMPDI}}_{\text {PL+PK}}$

Sensitivity: 0.63; Specificity: 0.80;		Predicted group	
Accuracy: 0.71; Threshold:0.54;		PL or PK	Normal
Actual group	PL or PK	170 (50, 120)	98 (22, 76)
	Normal	48 (16, 32)	196 (68, 128)

The numbers in parentheses represent the number of men and women.

**TABLE 5 table5:** Confusion Matrix When Discriminating Between PL and Normal Groups Using the Discrimination Threshold of ${\text {CMPDI}}_{\text {PL}}$

Sensitivity: 0.78; Specificity: 0.81;		Predicted group	
Accuracy: 0.80; Threshold: 0.39;		PL	Normal
Actual group	PL	111 (43, 68)	32 (11, 21)
	Normal	47 (15, 32)	197 (69, 128)

The numbers in parentheses represent the number of men and women.

**TABLE 6 table6:** Confusion Matrices When Discriminating Between (a) Oversampled PK and Normal and (b) Original PK and Normal Groups Using the Discrimination Threshold of CMPDI_PK_

(a)	Sensitivity: 0.87; Specificity: 0.84;		Predicted group	
	Accuracy: 0.85; Threshold: 0.48;		PK (Over-sampling)	Normal
	Actual group	PK (Over-sampling)	188	28
		Normal	39	205

(b)	Sensitivity: 0.81; Specificity: 0.84;		Predicted group	
	Accuracy: 0.84; Threshold: 0.48;		PK (Original)	Normal
	Actual group	PK (Original)	44 (9, 35)	10 (1, 9)
		Normal	39 (15, 24)	205 (69, 136)

The numbers in parentheses represent the number of men and women.

**TABLE 7:** Confusion Matrices When Discriminating Between (a) Oversampled PL $\times $PK and normal and (b) original PL $\times $PK and Normal Groups Using the Discrimination Threshold of ${\text {CMPDI}}_{\text {PL}\times \text { PK}}$

(a)	Sensitivity: 0.91; Specificity: 0.67;		Predicted group	
	Accuracy: 0.78; Threshold: 0.39;		PL and PK (Over-sampling)	Normal
	Actual group	PL and PK (Over-sampling)	193	20
		Normal	80	164

(b)	Sensitivity: 0.85; Specificity: 0.67;		Predicted group	
	Accuracy: 0.71; Threshold: 0.39;		PL and PK (Original)	Normal
	Actual group	PL and PK (Original)	60 (6, 54)	11 (2, 9)
		Normal	80 (27, 53)	164 (57, 107)

The numbers in parentheses represent the number of men and women.

**TABLE 8:** Discrimination Performances Between the Pain and Normal Groups Based on the CMPDI Values Derived Using the Voice Characteristics Controlled for Height and BMI

	PL or PK vs Normal	PL vs Normal	PK vs Normal	PL and PK vs Normal
Sensitivity	0.63	0.78	0.76	0.77
Specificity	0.80	0.79	0.84	0.64
Accuracy	0.71	0.79	0.83	0.67

**TABLE 9:** Sex Ratios and Mean Values (Standard Deviations) of the Attributes for Participants Under 65 Years of Age

	Normal Group	PL or PK	PL only	PK only	PL and PK
	Total number, 286	Total number, 219	Total number, 159	Total number, 22	Total number, 38
Men/Women	107/179	73/146	57/102	7/15	9/29
Age (years)	51.6 (9.5)	52.9 (8.5)	52.0 (8.7)	55.1 (7.8)	55.1 (7.7)
Weight (kg)	59.6 (12.0)	61.4 (12.3)	61.2 (12.8)	59.7 (9.2)	63.0 (11.4)
Height (cm)	162.0 (8.2)	161.8 (8.2)	162.5 (8.0)	161.6 (8.9)	159.3 (8.5)
BMI (kg/m^2^)	22.6 (3.6)	23.3 (3.7)	23.0 (3.7)	22.9 (3.1)	24.8 (3.6)

BMI = body mass index

The predominance of women can be noted in all groups. The sex ratio is more uneven in the PK and PL $\times $PK groups. In all groups, the results of the chi-square test ( $\chi ^{2}(4)=23.01;\ p< 0.001$) reveal a significant difference in the male-to-female ratio. As previously mentioned in Section II-E, two of the pairs of groups ((i) normal and PK and (ii) normal and PL $\times $PK groups) fulfill the conditions for applying the SMOTE, resulting in 216 and 213 samples in the PK and PL $\times $PK groups, respectively. In terms of age and weight, the analysis of variance indicates no significant differences between the mean values among the examined groups (age: $F(4,775)=1.83,\ p=0.12$; weight: $F(4,775)=0.72,\ p=0.58$). However, in terms of height and BMI, the analysis of variance indicates significant differences between the mean values among the examined groups (height: $F(4,775)=7.08,\ p< 0.001$; BMI: $F(4,775)=4.11,\ p< 0.01$). The results of the Tukey-Kramer test [25] indicate significant differences in height between the normal and PL $\times $PK groups ( $p< 0.001$), PL+PK and PL $\times $PK groups ( $p< 0.05$), PL and PK groups ( $p< 0.05$), and PL and PL $\times $PK groups ( $p< 0.001$), and significant differences in BMI between the normal and PL+PK groups ( $p< 0.05$) and normal and PL $\times $PK groups ( $p< 0.01$).

### B. Selection of Characteristics

As outlined earlier in Section II-E, for four of the pairs of groups ((a) normal and PL+PK, (b) normal and PL, (c) normal and PK, and (d) normal and PL $\times $PK groups), characteristics with absolute correlation coefficients of 0.1 or higher, determined from the vitality and MMSE scores, and characteristics with sex differences at a significance level of 5% were excluded. As a result, 1801 characteristics were obtained for the pair of PL+PK and normal groups, 1648 for the pair of PL and normal groups, 1482 for the pair of PK and normal groups, and 1837 for the pair of PL $\times $PK and normal groups. In the subsequent selection of characteristics using AUC, 512 characteristics were obtained for the pair of PL+PK and normal groups, 387 for the pair of PL and normal groups, 460 for the pair of PK and normal groups, and 457 for the pair of PL $\times $PK and normal groups. The selection of characteristics using AUC was performed on the data after oversampling by the SMOTE for the required pairs. Table 3 presents the details of the selected characteristics. Note that the mel-frequency, reported in Table 3, is a frequency that takes into account human auditory characteristics and is calculated by adjusting the original frequency to a scale that measures the pitch of a sound as perceived by humans (the mel scale [26]). Mel-frequency cepstral coefficients (MFCCs) [27] are Fourier coefficients of the mel-frequency spectrum.

### C. Regularized Logistic Regression Analysis

Principal component analysis was applied to the selected characteristics to obtain principal components with cumulative contribution rates of up to 80%. The analysis resulted in 51 principal components for the pair of PL+PK and normal groups, 59 for the pair of PL and normal groups, 43 for the pair of PK and normal groups, and 35 for the pair of PL $\times $PK and normal groups.

**FIGURE 3.**
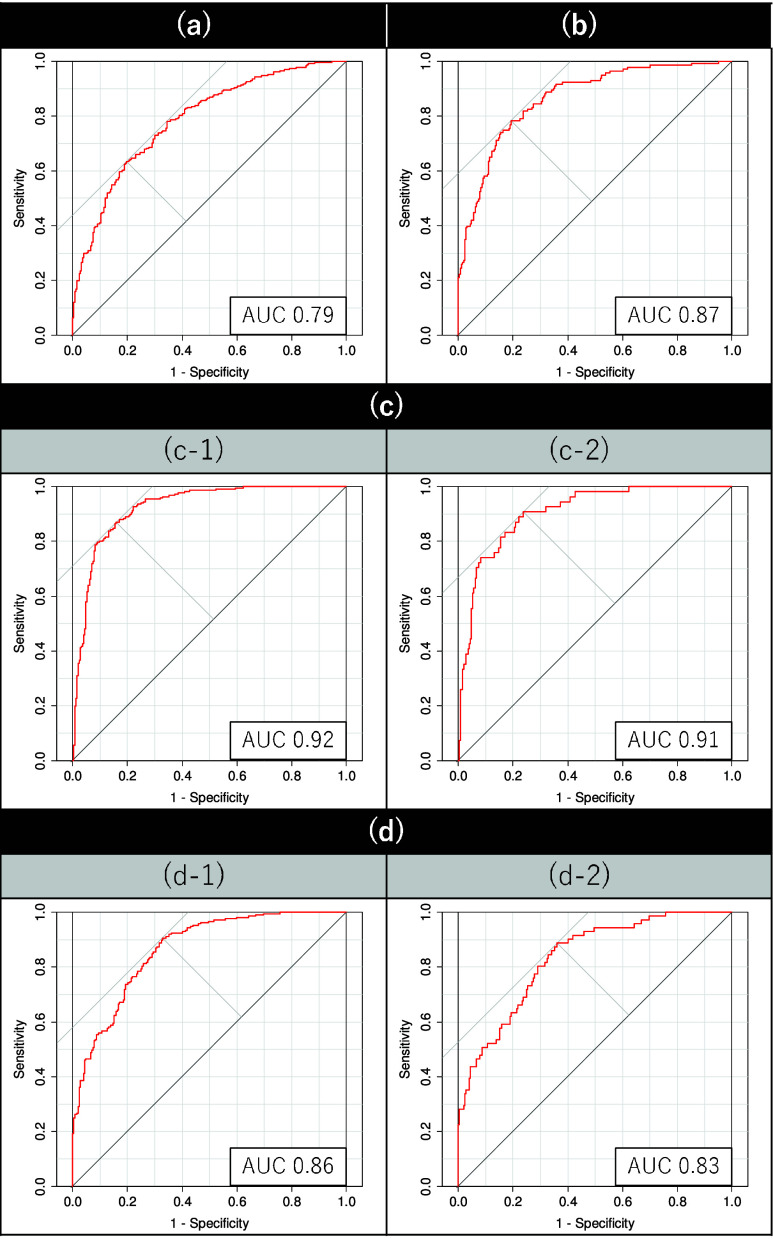
ROC curves for CMPDI values derived from the characteristics of the following groups: (a) PL+PK and normal, (b) PL and normal, (c-1) over-sampled PK and normal, (c-2) original PK and normal, (d-1) over-sampled PL $\times $PK and normal, and (d-2) original PL $\times $PK and normal groups. Sensitivity corresponds to the true positive rate, while $1-\text {Specificity}$ represents the false positive rate.

**FIGURE 4.**
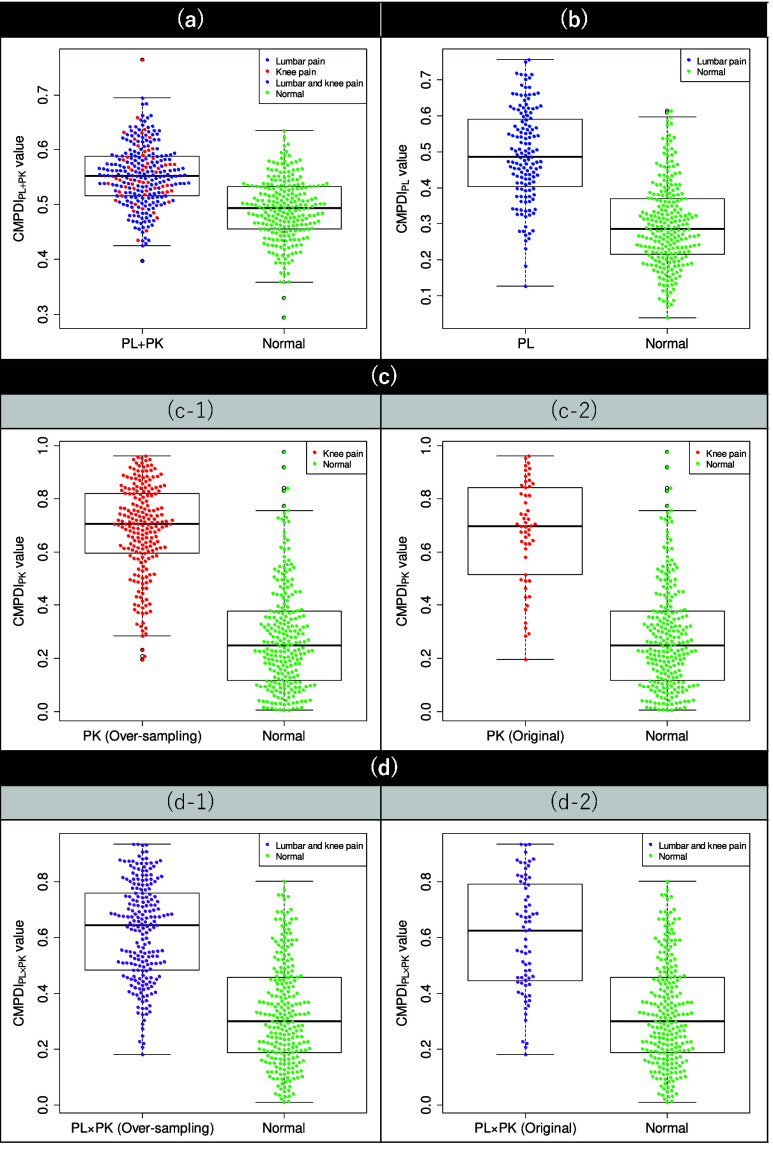
Distributions of CMPDI values for the pairs of the (a) PL+PK and normal, (b) PL and normal, (c-1) over-sampled PK and normal, (c-2) original PK and normal, (d-1) over-sampled PL $\times $PK and normal, and (d-2) original PL $\times $PK and normal groups.

**FIGURE 5.**
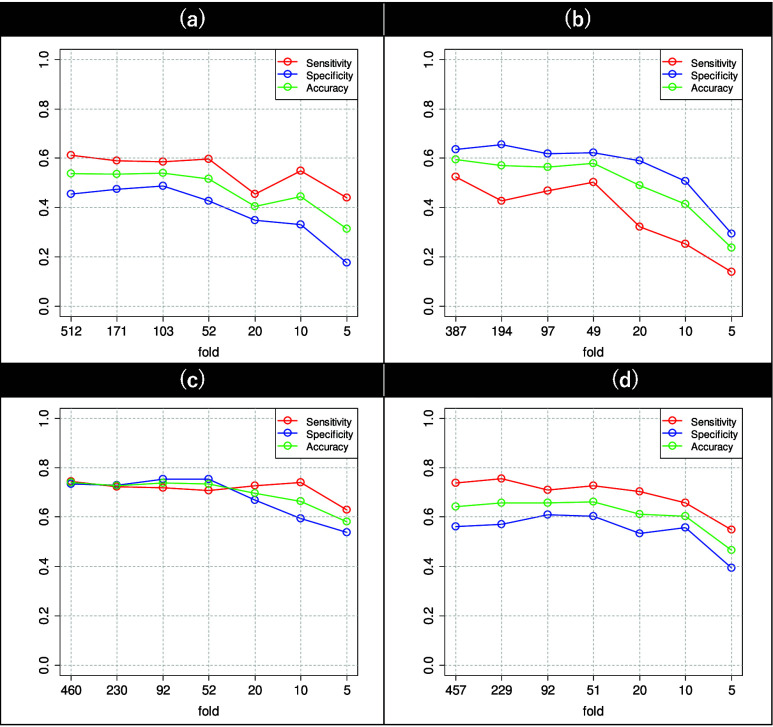
Results of *K*-fold cross-validation tests on the dataset of the pairs of the (a) PL+PK and normal, (b) PL and normal, (c) PK and normal, and (d) PL $\times $PK and normal groups. The vertical axis represents the ratio of sensitivity, specificity, and accuracy.

Using the regression coefficients, CMPDI was calculated using the following equation:

\begin{align*} \text { CMPDI}_{g\in G}& =\frac {1}{1+\exp {\left ({{-\beta _{g,0}-\sum ^{n_{g}}_{i=1}\beta _{g,i}PC_{g,i}}}\right)}} \\ G& =\left \{{{\text {PL+PK, PL, PK, PL}\times \text{PK}}}\right \} \tag {1}\end{align*}

where *G* is the index set of the pain groups; $n_{g}$ is the number of principal components in the dataset of the pair of normal and *g* groups with $g\in G$; $\beta _{g,0}$ is the intercept; $\beta _{g,i}$ is the regression coefficient for the *i*th principal component; $PC_{g,i}$ is the value of the *i*th principal component. The threshold value of ${\text {CMPDI}}_{g}$, used to discriminate between the normal and *g* groups, is the value that minimizes the BER corresponding to the ${\text {CMPDI}}_{g}$ values calculated for subjects using Eq. (1). The confusion matrices obtained after discrimination based on the collected data between the normal and *g* groups using the threshold values of ${\text {CMPDI}}_{g}$ are presented in Tables 4 [Table table5][Table table6]–7. The discrimination performance of the ${\text {CMPDI}}_{g}$ based on the collected data is presented in Figs. 3 and 4. The generalizability of the ${\text {CMPDI}}_{g}$ is presented in Fig. 5.

In Fig. 4, the average ${\text {CMPDI}}_{g}$ values derived for the pair of *g* and normal groups show a significant difference at the 1% significance level in a Welch’s t-test, which did not assume equal variance for all *g* groups.

The calculated recall rates for each gender in the pain and normal groups of the confusion matrices in Tables 4 [Table table5][Table table6]–7 are as follows: (i) for the pair of PL+PK and normal groups, the results were (man, woman) = (0.69, 0.61) in the PL+PK group, (man, woman) = (0.81, 0.80) in normal group; (ii) for the pair of PL and normal groups, the results were (man, woman) = (0.80, 0.76) in the PL group, (man, woman) = (0.82, 0.80) in the normal group; (iii) for the pair of PK and normal groups, the results were (man, woman) = (0.90, 0.80) in the PK group, (man, woman) = (0.82, 0.85) in the normal group; and (iv) for the pair of PL $\times $PK and normal groups, the results were (man, woman) = (0.75, 0.86) in the PL $\times $PK group, (man, woman) = (0.68, 0.67) in the normal group. Note that no significant differences were observed based on the recall rates (≤ 0.1) between men and women in any of the examined pairs.

As shown in Fig. 5, when the number of folds is equal to the number of subjects in the entire dataset, the evaluation represents a leave-one-out cross-validation approach. Notably, for all pairs of groups the sensitivity and specificity remain at an almost constant value until approximately 50 folds and then decrease as the number of folds decrease.

### D. Effect of Height and BMI on Voice Characteristics

**FIGURE 6.**
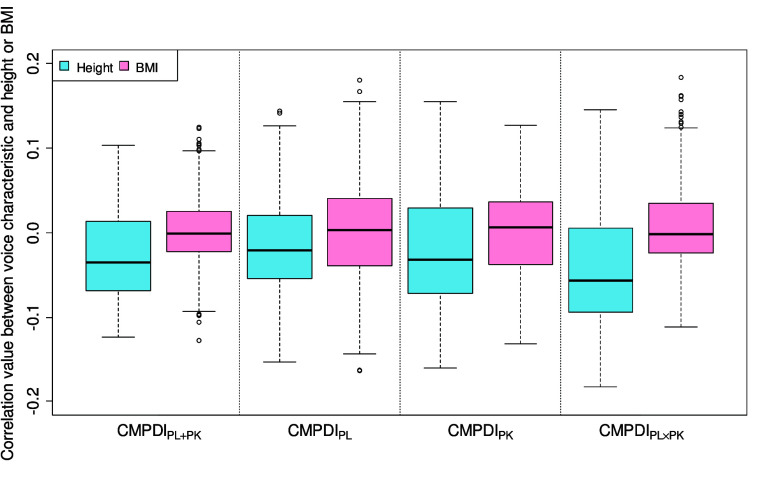
Distribution of correlation values between the voice characteristics included in deriving the CMPDI values and heights or BMIs of the subjects in each group.

Figure 6 shows the distribution of correlation values between the voice characteristics included in deriving the CMPDI values and heights or BMIs of the subjects in each group. In Fig. 6, based on the heights of the subjects, negative correlation values can be observed to dominate the CMPDI values. In Fig. 6, based on the BMIs of the subjects, a correlation value of approximately 0 can be observed to dominate the CMPDI values, irrespective of the group. The maximum of absolute correlation values between voice characteristics and heights or BMIs of the subjects in all groups did not exceed 0.2. Furthermore, to quantitatively investigate the influence of height and BMI on the CMPDI values, the voice characteristics included in deriving the CMPDI were regressed on height and BMI. The voice characteristics were controlled to be uncorrelated with height and BMI by calculating the residuals by subtracting the predicted voice characteristics based on height and BMI from the actual voice characteristics. To adjust the range of the original voice characteristics, the average value evaluated from the original voice characteristics was added to the residuals. Subsequently, the adjusted residuals were utilized for evaluating the CMPDI values. Finally, the discrimination performances between the pain and normal groups were evaluated, and the results are shown in Table 8. No significant differences in the sensitivity and specificity can be observed before and after incorporating the height/BMI-based control to the voice characteristics.

## IV. Discussion

To the best of our knowledge, this is the first study to detect pain using the changes in voice prosody. The threshold for recognizing a physical stimulus as pain varies among individuals. Therefore, assessing whether the proposed index correctly detects and identifies an individual feeling pain is important than the assessing severity of the physical stimulation.

Physical pain often causes a decline in ADL and QOL. A decline in ADL and QOL in elderly people increases the risk of prognosis, such as needing nursing care or death, inhibits social participation, and increases social isolation, often causing depression and dementia [18], [19]. Therefore, global preventive measures for combatting physical pain, especially chronic pain in the musculoskeletal system, are urgently needed. Given the global trend of population aging, with Japan reporting the highest aging rate in the world, this study focused on elderly people, recognizing the need to understand and address the challenges and opportunities presented by an aging population both in Japan and worldwide.

As listed in Table 2, out of 156 men and 356 women, lumbar pain is detected in approximately 40% men and 43% women (including combined knee pain), whereas knee pain is detected in approximately 12% men and 30% women (including combined lumbar pain). The prevalence of lumbar pain is considerably higher than that of knee pain in men. In contrast, no significant difference exists in the number of cases of lumbar or knee pain among women. The obtained results are consistent with the ranking order of symptoms in the sex-based pain groups in Japan [1].

Note that mental changes and neurological abnormalities caused by illness can manifest as voice problems, as the nerves controlling the vocal cords are directly affected by neurological conditions. The voice characteristics-based method discussed in this study focuses on analyzing the voice changes caused by abnormalities in the vocal cords that cannot be controlled by individuals. Based on the results listed in Tables 4 [Table table5][Table table6]–7 and shown in Fig. 3, the derived CMPDI values obtained through learning show an accuracy rate of more than 70% for all training data in all datasets, indicating that the derived CMPDI values effectively discriminate between each pain group and the normal group, with an AUC of approximately 0.8 or higher. In Fig. 4, although a lot of overlap can be observed in the distributions of CMPDI values between the pain and normal subjects, the results of the Welch’s t-test indicate significant differences in their mean values.

In Table 6, the dataset obtained from the pair of PK and normal groups can be observed to exhibit relatively high sensitivity and specificity, with greater than 80% accuracy. Thus, the subjects suffering from knee pain can be concluded to have more distinctively identifiable voice characteristics than those of the normal subjects. In addition, oversampling using SMOTE, which is a preprocessing step of learning, yielded the same number of pseudo-samples for the PK and normal groups without any extreme bias in either the sensitivity or specificity of the learning results, suggesting that oversampling has little to no effect on learning. In Fig. 5, according to the cross-validation tests, both sensitivity and specificity have approximately 70% accuracy until about 50 folds, after which specificity declines while sensitivity remains at approximately 70% until 10 folds. Therefore, the possibility of overlearning is low when utilizing all training data in the dataset obtained from the pair of PK and normal groups.

In Table 7, the sensitivity is over 80% even for the dataset obtained from the pair of PL $\times $PK and normal groups, further confirming that the subjects with knee pain have more distinctively identifiable voice characteristics than those of the normal subjects. When comparing the specificities observed in similar studies, the specificity derived using the dataset of this study can be observed to be relatively low, possibly because the subjects in the PL $\times $PK group experienced lumbar pain in addition to knee pain. The specificity was observed to decrease when learning included voice characteristics that have specific values in the pain group but vary widely in the normal group. Possibly, the lumbar pain-specific characteristics exhibit a similar decrease in specificity. According to the sensitivity and specificity of the learning results, it appears that oversampling using SMOTE weakly affects learning outcomes. According to the results of the cross-validation tests, shown in Fig. 5, the sensitivity and specificity are approximately 70% and 60%, respectively, up to approximately 50 folds, after which the sensitivity declines while the specificity remains at approximately 60% up to 10 folds. Therefore, the possibility of overlearning can be considered low for all training data in the dataset obtained from the pair of PL $\times $PK and normal groups.

In Table 5, the dataset obtained from the pair of PL and normal groups can be observed to exhibit a slightly lower sensitivity than those exhibited by the pairs of PK and normal groups and PL $\times $PK and normal groups. The dataset obtained from the pair of PL and normal groups exhibits a higher specificity than that exhibited by the pair of PL $\times $PK and normal groups, contradicting the speculation that the specificity would be low if lumbar pain is a common factor between the groups. However, the results of cross-validation tests in Fig. 5 show that both sensitivity and specificity are significantly lower, suggesting overlearning for the entire training dataset. The selected characteristics in the pre-learning process were converted into principal components using principal component analysis. Notably, the dataset obtained from the pair of PL and normal groups exhibits the highest number of principal components, with a lower number of voice characteristics per principal component, implying that only a few characteristics exhibit similar distributions or that the variation between characteristics is large. A large variation between voice characteristics can be anticipated to result in a complex composition and a relatively high expressiveness of the CMPDI, making possibility of overlearning high and increasing the detection inaccuracy for unknown datasets. Comparing the results of cross-validation tests in Fig 5, the specificity of the dataset obtained from the pair of PL and normal groups can be observed to match the dataset obtained from the pair of PL $\times $PK and normal groups up to approximately 50 folds, with a sensitivity of approximately 50%, implying that the lumbar pain-specific characteristics vary widely among the subjects in the pain group. The characteristics of normal subjects also vary widely; thus, the voice characteristics of subjects with lumbar pain can be concluded to be similar to those of normal subjects. Following the discussion on the pair of PL $\times $PK and normal groups, the inclusion of knee pain in addition to lumbar pain can be concluded to suppress the variability of the characteristics exhibited by the pain group, increasing the sensitivity of pain detection.

According to Table 4, among all datasets, the dataset obtained from the pair of PL+PK and normal groups exhibits the lowest sensitivity, possibly because the number of subjects with lumbar pain is significantly greater than the number of subjects with knee pain; thus, the voice characteristics associated with knee pain could not be completely established. However, the high specificity of the dataset obtained from the pair of PL+PK and normal groups cannot be explained by the above discussion. By contrast, the results of cross-validation tests in Fig. 5 show a significant decrease in specificity, indicating overlearning for the entire training dataset. The cross-validation tests show that the sensitivity of the dataset remains at approximately 60% up to approximately 50 folds, and the deviation of sensitivity from that of the entire training dataset is small. The specificity remains less than approximately 50% for all folds, possibly because of a large number of subjects with lumbar pain.

In conclusion, subjects with knee pain have more distinctively identifiable voice characteristics than those of normal subjects, whereas subjects with lumbar pain exhibit voice characteristics similar to those of normal subjects. However, it is unclear why the subjects with lumbar pain have voices similar to those of normal subjects. Possibly, the subjects with lumbar pain experience relatively milder symptoms. Alternatively, differences in pain perception and experience can exist between knee pain and lumbar spine pain. Lumbar spine pain is a multifactorial condition caused by many factors, including morphological abnormalities of the intervertebral disc and vertebral body, as well as alignment abnormalities and osteoporosis-related microfractures [28]. Lumbar pain is also strongly influenced by mental health, making identification of its characteristics more difficult as compared to those of knee pain. Future studies will be designed to investigate the different characteristics of lumbar pain and knee pain by further examining the cohort.

According to Table 3, among all datasets, only the dataset obtained from the pair of PK and normal groups exhibits a large number of mel-frequency power spectrum-related characteristics. Therefore, knee pain can be considered to have a large effect on the mel-frequency power spectrum. In the dataset obtained from the pair of PL and normal groups, in addition to the differences in the mel-frequency power spectrum, significant differences can be observed in the spectral power-related characteristics, i.e. characteristics related to the short-time frequency power of the voice, and MFCC-related characteristics, with the selection rate being higher than that of the dataset obtained from the pair of PK and normal groups. Therefore, lumbar pain can be considered to significantly affect the spectral power- and MFCC-related characteristics. Furthermore, the dataset obtained from the pair of PL $\times $PK and normal groups exhibits a strongly distinctive trend in the characteristics related to lumbar pain rather than knee pain. The dataset obtained from the pair of PL+PK and normal groups exhibits almost the same trend in the selected characteristics as the dataset obtained from the pair of PL and normal groups, indicating the dominance of lumbar pain-related characteristics. The observed differences among the groups can be attributed to the fact that the PL+PK group has a large number of subjects with lumbar pain. During data analysis, spectrum-related characteristics (spectral and mel-frequency spectral powers) were selected across a wide range of bands from low to high; however, determining the frequency band that responds to pain was difficult. Therefore, a future task is to identify the voice characteristics that correspond to the pain-related physiological changes. Numerous studies have shown that pain disrupts the balance between sympathetic and parasympathetic nerves, causing the heart rate to fluctuate [29], [30]. The vocal cords, like the heart, are innervated by branches of the vagus nerve, specifically the recurrent laryngeal nerve and superior laryngeal nerve, making it plausible that similar changes or conditions affecting the vagus nerve can impact vocal cord function. Among the selected voice characteristics, proportions of the characteristics related to the amplitude of the speech-time waveform and F_0_ are low in all datasets. In this study, a few characteristics related to the amplitude of the speech-time waveform were selected because the voice was normalized by volume. Since the characteristics that significantly differ between men and women were also excluded, some characteristics related to F_0_ were selected. In addition, from the recorded long vowel sounds, a few characteristics related to the voiced sound probability were selected.

In Table 2, in the pain and normal groups, the predominance of women over men can be noted. If the sex ratio had been similar between the pain and normal groups, the effect of the sex ratio on the CMPDI would have been negligible; however, since the sex ratio can be observed to differ significantly between the pain and normal groups, the impact of the sex ratio on the derived CMPDI values can be expected to be significant. Consequently, the derived CMPDI values may be more strongly influenced by gender than by pain. In this study, the sex ratio in some of the pain groups differed significantly from that in the normal group. Therefore, the voice characteristics with significant differences between men and women were excluded while deriving the voice index. The gender-based recall rates in the pain and normal groups, as shown in the confusion matrices in Tables 4 [Table table5][Table table6]–7, demonstrate that the difference in the recall rates between men and women is ≤ 0.1; therefore, the exclusion of gender-based characteristics can be concluded to not affect the determined CMPDI values. In conclusion, gender is not a discriminative factor between the pain and normal groups.

In this study, the elderly subjects mostly complained of lumbar pain, making the chances of detecting lumbar pain high. Our analysis revealed that the voice characteristics of subjects with lumbar pain were similar to those of normal subjects, possibly because lumbar pain is accompanied by several complex and diverse symptoms. Many potential confounding factors may contribute to lumbar pain; thus, evaluating other parameters apart from voice may help differentiate subjects with lumbar pain from normal subjects. In addition to voice characteristics, the evaluation of various parameters for a large number of subjects is required for correctly analyzing lumbar pain. Note that cohort studies often require a large number of subjects because they involve following a group over time to observe the incidence of a specific outcome, which may be rare, and to ensure statistical power and generalizability of findings. However, in cohort studies, older adults are at a higher risk of mortality during the study period, leading to attrition and potentially biasing the results. Another limitation is that frailty and health decline can make it difficult for older adults to continue participating in the study, leading to loss of follow-up. Based on the data presented in Table 2, the rate of complaints of only lumbar pain in older adults who participated in this study is approximately 28%. Based on the data presented in Table 9 in the Appendix, the rate of complaints of only lumbar pain in participants under 65 years of age who participated in the ROAD study is approximately 31%, which is slightly higher than that determined for older adults. In conclusion, analysis based on CMPDI is more suitable for detecting lumbar pain in young adults. Although voice characteristics change with age, no significant age-related differences can be observed among any of the groups in listed Table 2; therefore, voice characteristics were not excluded based on age. However, the proposed CMPDI values are not appropriate indices for detecting musculoskeletal pain in young adults, indicating the need for reconstructing the CMPDI for an accurate detection of musculoskeletal pain. Furthermore, we believe that the proposed CMPDI can also be made applicable on individuals, with different cultural backgrounds and speaking different languages other than Japanese.

Height and BMI may affect the voice characteristics. Therefore, height and BMI may be confounding factors that might affect the findings of this study. Based on heights of the subjects, significant differences were observed between the normal and PL $\times $PK groups. Based on BMIs of the subjects, significant differences were observed between the (i) normal and PL+PK groups and (ii) normal and PL $\times $PK groups. The differences in height/BMI may also affect discrimination performances between the pain and normal groups based on the CMPDI values. In Fig. 6, no voice characteristics included in deriving the CMPDI values can be observed to be significantly correlated with height and BMI in each group. To account for the influence of height/BMI on voice characteristics, we have incorporated some controls to the voice characteristics based on height/BMI. We evaluated the discrimination performance of each CMPDI using the controlled voice characteristics. Comparing the values listed in Table 8 with those listed in Tables 4 [Table table5][Table table6]–7, the sensitivities evaluated for the controlled values of CMPDIs for PK and PL $\times $PK detections can be observed to be slightly lower; however, no significant differences in sensitivity and specificity can be observed among various groups. Based on the results of this study, the impact of height and BMI on pain detection and evaluation can be considered as negligible. In conclusion, pain can be efficiently evaluated using the proposed voice characteristics-based index, CMPDI, without the need for implementing controls arising from heights or BMIs of the subjects.

This study evaluated pain as a binary classification (presence or absence of pain) utilizing voice characteristics. Quantitative assessment of pain intensity can also be performed using scales, such as VAS or NRS. However, scope of the present study is limited to CMPDI-based analysis. Since the pain intensity is not evaluated using any scales in this cohort study, constructing a numerical prediction index using the existing data is not possible. In future cohort studies, we will definitely consider incorporating a questionnaire that numerically evaluates pain intensity, construct a voice index to predict pain, and analyze correlations between various pain parameters extracted from the voice-based data.

In this study, cross-validation tests (refer to Fig. 5 for the results) were used to assess the generalizability of the derived CMPDI values. Typically, cross-validation and independent validation tests are performed on datasets different from the training dataset to assess how well a model generalizes to unseen data. Notably, using both an independent validation dataset and cross-validation for data validation provides a more robust and comprehensive assessment of the performance and generalization ability of a model. However, at this stage, we are unable to create an independent validation dataset and perform the required validation because frequent large cohort surveys are difficult to conduct, and obtaining new datasets requires a considerable amount of time and effort. In our next ROAD study, we anticipate to validate the CMPDI against an independent validation dataset. However, in the presented cohort study, the responses of subjects are almost same; however, the analyzed data can be considered new because of the several-year interval between data points and changing voice characteristics.

Several studies have combined physiological indices other than voice characteristics using learning methods to detect and evaluate pain [31]. Most of the existing studies focus on pain detection via thermal or electrical stimulation. Only a few studies have attempted to detect chronic pain in the musculoskeletal system. In the experiment conducted by Jiang et al. [32], healthy subjects were given thermal and electrical stimulation for a few seconds on their fingers, and then the heart rate, respiratory rate, and skin electrodermal response of the subjects were measured and facial electromyogram monitored. Based on the results obtained after simulation, Jiang et al. proposed a method to predict pain intensity. Pain intensity was evaluated using a VAS when the subject reported a fairly unpleasant level of pain and intolerable pain during stimulation. An unpleasant level of pain was labeled as mild pain and an intolerable pain was labeled as moderate/severe pain. A no pain label was also included to label subjects in a painless state few seconds before the stimulation. A classifier discriminating severities of pain was created by learning physiological indices, such as heart rate, respiratory rate, skin electrodermal response, and facial electromyogram, using a neural network. The classification accuracy of the pain detection method proposed by Jiang et al. was approximately 71%. Aung et al. [33] examined subjects with chronic lumbar pain by recording their facial expressions while making them perform various exercises. Lumbar pain was detected by assessing the video frames for facial expressions indicating pain. Furthermore, Aung et al. used a linear support vector machine to learn about feature vectors extracted from facial images to identify frames containing pain-related facial expressions. The discrimination performance of the pain detection method proposed by Aung et al. was approximately 0.64 as per AUC. Comparing the previously reported pain detection methods with our method, it can be mentioned that the accuracy of the analysis performed by Jiang et al. is comparable to that of the cross-validation tests performed on the CMPDI values evaluated for knee pain; however, a direct comparison of the results is difficult because the types of pain are different. Notably, the accuracy of the analysis performed by Aung et al. is comparable to that of the cross-validation tests performed on the CMPDI values evaluated for lumbar pain. To summarize, pain can be detected with accuracy utilizing physiological indices other than voice characteristics. However, the existing non-invasive pain detection methods require special equipment and are not easy to perform. Contrastingly, voice characteristics-based pain detection method does not require special equipment and can be easily performed, making the method proposed in our study superior to other existing non-invasive methods. With the widespread use of wearable technology, behavioral and/or physiological functions such as physical activity, heart rate, heart rhythm, and sleep heart rate can be measured relatively easily. The integration of behavioral and/or physiological attributes with voice characteristics can enable the establishment of other pain detection indices. The development of more accurate pain detection indices can significantly enhance the ability to assess patient discomfort in real-time, leading to more effective pain management strategies.

The research findings of this study can be translated into clinical practice by utilizing the voice index as an auxiliary tool for clinical detection of chronic musculoskeletal pain, especially in elderly individuals who have trouble communicating. Elderly people often do not actively complain about their pain because of their stoicism and prejudices related to pain [6]. The proposed voice index-based pain detection method can be viewed as an ethical way to approach sick elderly individuals who are suffering from dementia-like medical conditions and are unable to verbally communicate pain. The proposed pain detection method can also be utilized to remotely examine elderly people who live in depopulated areas and have difficulty visiting hospitals. The proposed method can also be used as a continuous monitoring tool to identify patients whose pain persists to the point where it reaches a state of constant sympathetic dominance, requiring urgent medical treatment. In the future, we anticipate introducing the proposed voice index in actual clinical settings and further examining the accuracy of the proposed voice index-based pain detection method.

## V. Conclusion

This study proposed an index to discriminate between normal subjects and subjects with chronic musculoskeletal pain based on the voice characteristics of elderly people 65 years of age or older. A large-scale population-based cohort study was conducted, and the voices of the subjects were recorded. The voice characteristics of the subjects were extracted and evaluated using the openSMILE software. The obtained datasets were categorized into pain-related and normal groups based on the body part (pain in the lumbar, knee, or both) for which pain was reported. For each dataset, voice characteristics related to depression and dementia were excluded, and the characteristics that significantly differed between men and women were also excluded. The principal component analysis was performed to organize the characteristics with similar properties into several principal components. A regularized logistic regression analysis was performed using the determined principal components as explanatory variables. Consequently, a voice index that could discriminate between the pain and normal groups was derived. Among training datasets, the dataset obtained using the voice characteristics of subjects with knee pain exhibited a sensitivity, specificity, and accuracy rate of more than 80%. The sensitivity, specificity, and accuracy rate remained above 70% up to approximately 50 folds even after cross-validation of the training data. The voice index derived using the dataset obtained from the voice characteristics of subjects with lumbar pain was not significant enough to discriminate between subjects with lumbar pain and normal subjects. The results of this study suggest that pain-related changes in voice may vary depending on the affected area, leaving room for further study. The voice characteristics-based pain detection index proposed in this study has the potential to quantitatively assess pain, which is otherwise a subjective sensation.

## Appendix

See Table 9.
